# Does birth by cesarean section affect children’s academic performance and intelligence?

**Published:** 2023-04

**MARCH 22, 2023 -** In a study of Danish children born between 1978–2000, chances of graduating from lower and upper secondary education were significantly lower for children born by cesarean section (CS). However, differences in grade point averages and intelligence scores were very small. The study, which is published in Acta Obstetricia et Gynecologica Scandinavica, also found that males born by CS had a lower likelihood of appearing before a conscription board for drafting into the military.

In Denmark, most students are 6–16 years old while in lower secondary education (LSE) and 16–17 years old when initiating upper secondary education (USE). Also, all Danish male citizens must appear before a Danish conscription board for military or civil service, unless one of the board’s doctors declares the person unfit for service prior to the examination.

“Cesarean section is a fairly common procedure. Luckily children born by cesarean section do not seem to perform less in the Danish educational and conscription system compared with children born by vaginal delivery; however, they do seem to have lower chances of attending education and conscription,” said first author Agnes Kielgast Ladelund, MD, of Herlev Hospital in Denmark. “These are interesting results, relevant for the nationwide discussion of pros and cons concerning cesarean section.”

***URL upon publication: https://onlinelibrary.wiley.com/doi/10.1111/aogs.14535***

***Full citation:** “Association of birth by cesarean section with academic performance and intelligence in youth: A cohort study.” Agnes K. Ladelund, Julie A. Slavensky, Frederik J. Bruun, Emilie Pi Fogtmann Sejer, Erik Lykke Mortensen, Steen Ladelund, Ulrik S. Kesmodel. Acta Obstet Gynecol Scand; Published Online: 22 March 2023 (DOI: 10.1111/aogs.14535).*

*Copyright © 2019 The Cochrane Collaboration. Published by John Wiley & Sons, Ltd., reproduced with permission.*

